# Bridging the gap: exploring healthcare professionals' perceptions and barriers towards cervical cancer screening services uptake amongst women in rural communities

**DOI:** 10.1186/s43046-026-00346-x

**Published:** 2026-04-06

**Authors:** Azubuike Amadi, Muili Lawal, Hafiz Khan, Sharry Japhet

**Affiliations:** 1 College of Nursing, Midwifery and Health, University of West London, London, United Kingdom; 2 Department of Community Medicine, Rivers State University, Port Harcourt, Nigeria; 3 Department of drug evaluation and research, National Agency for food and drug administration and control, Abuja, Nigeria

**Keywords:** Colposcopy, Cervical cancer screening, Rural healthcare, Barriers, Healthcare professionals' perceptions

## Abstract

**Background:**

Cervical cancer remains a significant public health concern, particularly in rural communities where access to screening and diagnostic services is limited. Despite being preventable through HPV vaccination and early detection via screening, cervical cancer continues to pose substantial health challenges in resource-limited settings. Colposcopy, a crucial diagnostic procedure for women with abnormal screening results, faces various implementation challenges in rural healthcare settings.

**Objective:**

This study explores healthcare professionals' perceptions, experiences, and identified barriers affecting cervical cancer screening services uptake among women in rural communities, with particular focus on colposcopy service delivery challenges and potential improvement strategies.

**Methods:**

Two qualitative focus group discussions were conducted with healthcare professionals (*n* = 18 total; FGD1: *n* = 9, FGD2: *n* = 9) involved in cervical cancer screening services in rural areas. Participants included obstetrician-gynaecologists, general practitioners, and healthcare providers with experience in colposcopy services. Data were analysed using thematic analysis to identify key themes and patterns.

**Results:**

Healthcare professionals identified multiple interconnected barriers affecting cervical cancer screening services uptake, including: (1) limited awareness and cultural beliefs among rural women, (2) infrastructural challenges including equipment shortages and power supply issues, (3) workforce limitations and inadequate training, (4) accessibility and transportation barriers, and (5) security concerns in rural areas. Despite these challenges, professionals acknowledged the critical importance of colposcopy in early cervical cancer detection and prevention.

**Conclusions:**

Based on healthcare professionals' experiences in this study of three local government areas in Rivers State, successful implementation of cervical cancer screening services in rural communities requires a multi-faceted approach addressing infrastructure development, continuous professional training, community education programmes, and policy support. Healthcare professionals emphasise the need for sustained commitment from government and non-governmental organisations to bridge existing gaps in cervical cancer prevention services.

## Introduction

Cervical cancer represents one of the most preventable forms of cancer, yet it continues to pose a significant threat to women's health globally, particularly in low and middle-income countries [8]. The disease's preventable nature stems from its well-understood natural history, with identifiable precancerous lesions that can be detected and treated before progression to invasive cancer [52]. Colposcopy, as a specialised diagnostic procedure, plays a pivotal role in the evaluation of abnormal cervical cytology and the early detection of cervical intraepithelial neoplasia (CIN) and invasive cervical cancer [57].

Human papillomavirus (HPV) infection is the primary etiological factor for cervical cancer, with persistent high-risk HPV types responsible for virtually all cases of cervical cancer [18, 56]. HPV vaccination represents the cornerstone of primary prevention, while screening serves as secondary prevention for detecting and treating precancerous lesions before they progress to invasive cancer [52]. The integration of both vaccination and screening is essential for comprehensive cervical cancer prevention [59].

In Nigeria, cervical cancer ranks as the second most common cancer among women, with an estimated age-standardised incidence rate of 23.7 per 100,000 women [25, 3]. The burden is disproportionately higher in rural communities, where access to preventive healthcare services, including cervical cancer screening, remains significantly limited [9, 21]. This disparity is particularly concerning given that rural women often present with advanced-stage disease, resulting in poorer outcomes and higher mortality rates [19].

Nigeria's healthcare system is structured across three tiers: primary, secondary, and tertiary levels. However, the distribution of healthcare professionals, particularly specialists, is heavily skewed towards urban areas. Rural communities experience severe shortages of trained healthcare providers, with most obstetrician-gynaecologists concentrated in urban tertiary hospitals [2, 3]. This geographic maldistribution creates significant barriers to accessing specialised services like colposcopy in rural settings.

HPV vaccination in Nigeria has made recent progress despite implementation challenges. Nigeria officially introduced the single-dose HPV vaccine into the National Programme on Immunisation (NPI) in October 2023, targeting girls aged 9–14 years [42, 60, 23]. Prior to national introduction, pilot vaccination programmes were conducted in several states to assess feasibility and acceptability [43]. However, nationwide coverage remains limited due to cost, supply chain challenges, logistical barriers in reaching all eligible girls, and low awareness among both healthcare providers and the general population [7].

Nigeria does not currently have a fully organized, population-based national cervical cancer screening program [20]. Screening services are largely opportunistic and facility-based, concentrated primarily in tertiary hospitals in urban areas. There is no systematic recall or follow-up system, and many women never receive screening throughout their lifetime [32]. Unlike some organised breast cancer screening initiatives in select states, cervical cancer screening lacks coordinated national-level implementation, contributing to late-stage presentation and poor outcomes [31].

The implementation of effective cervical cancer screening programmes in rural settings faces numerous challenges, ranging from inadequate healthcare infrastructure to cultural barriers and limited healthcare workforce [61, 58]. Understanding healthcare professionals' perspectives on these challenges is crucial for developing targeted interventions that can improve screening uptake and ultimately reduce cervical cancer morbidity and mortality in underserved populations.

Colposcopy, introduced by Hans Hinselmann in 1925, has evolved to become an essential component of cervical cancer prevention programmes worldwide [54]. The procedure allows for magnified visualisation of the cervix, enabling healthcare providers to identify abnormal areas that may not be visible to the naked eye and to guide targeted biopsies when necessary [38]. However, the successful implementation of colposcopy services requires adequate training, appropriate equipment, and supportive healthcare infrastructure, resources that are often limited in rural healthcare settings.

This study aims to explore healthcare professionals' perceptions, experiences, and identified barriers affecting cervical cancer screening services uptake among women in rural communities focusing on colposcopy, with the goal of informing evidence-based strategies for improving cervical cancer prevention services in underserved areas.

## Literature review

### Cervical cancer screening in resource-limited settings

Cervical cancer screening programmes have demonstrated remarkable success in reducing disease incidence and mortality in high-income countries [49]. However, implementing similar programmes in resource-limited settings presents unique challenges that require adapted approaches and innovative solutions [51]. The World Health Organisation (WHO) has emphasised the importance of context-specific screening strategies that consider local resources, infrastructure, and cultural factors [59].

In sub-Saharan Africa, where cervical cancer rates remain among the highest globally, screening coverage is often inadequate, with many women never receiving screening services throughout their lifetime [14]. Rural communities face additional challenges, including geographic isolation, limited transportation options, and reduced availability of skilled healthcare providers [45].

### Role of colposcopy in cervical cancer prevention

Colposcopy serves as a crucial bridge between primary screening (cytology or HPV testing) and definitive treatment of precancerous lesions [5]. The procedure's diagnostic accuracy depends heavily on the colposcopist's experience and training, emphasising the importance of standardised training programmes and continuous professional development [12].

Emerging developments in colposcopy technology, including digital imaging and artificial intelligence-assisted interpretation, show promise for future applications. However, as of 2026, these technologies remain largely in research and development phases without adequate validation for routine clinical practice in resource-limited settings [29, 62]. Current implementation efforts should focus on ensuring access to standard, well-validated colposcopy equipment rather than unproven technological solutions.

### Barriers to cervical cancer screening in rural communities

Previous research has identified multiple barriers to cervical cancer screening in rural communities, including patient-level factors (knowledge, attitudes, cultural beliefs), healthcare system factors (access, availability, quality), and structural factors (transportation, costs, geographic distance) [4, 30]. Healthcare provider perspectives on these barriers are essential for developing comprehensive interventions that address system-level challenges.

Cultural and religious beliefs significantly influence women's decisions regarding gynaecological examinations and screening procedures [1]. Understanding and addressing these cultural factors requires culturally sensitive approaches that involve community leaders and traditional healers in health education initiatives [46].

## Methodology

### Study design

This qualitative study employed a focus group discussion approach to explore healthcare professionals' perceptions and experiences regarding colposcopy screening services in rural communities. The qualitative methodology was chosen to enable in-depth exploration of complex issues and to capture the nuanced perspectives of healthcare providers working in challenging environments.

### Participants and recruitment

#### Recruitment strategy and data saturation

Healthcare professionals were recruited through purposive sampling from rural healthcare facilities across three local government areas in Rivers State, Nigeria [26]. The recruitment approach involved initial contact with facility administrators and department heads who identified eligible participants based on the inclusion criteria. Healthcare facilities approached included primary healthcare centres, general hospitals, and rural outreach centres providing women's health services [48].

Inclusion criteria required participants to have: (1) been trained and at least one year of experience providing healthcare services in rural communities, (2) direct involvement in cervical cancer screening programmes, and (3) current employment in a rural healthcare facility. Exclusion criteria included healthcare providers working exclusively in urban centres or those without direct experience in women's reproductive health services [17].

Two focus group discussions were conducted with a total of eighteen healthcare professionals (*n* = 18 total; FGD1: *n* = 9, FGD2: *n* = 9), including obstetrician-gynaecologists (*n* = 4), general practitioners (*n* = 9), nurse practitioners (*n* = 3), and healthcare coordinators (*n* = 2) with varying levels of experience in cervical cancer screening and colposcopy services. Data saturation was assessed across both focus group discussions. The first focus group (*n* = 9) generated rich initial themes, while the second focus group (*n* = 9) was conducted to verify and expand upon these findings. By the conclusion of the second discussion, no new substantive themes emerged, and existing themes were well-saturated with diverse perspectives from participants representing different professional backgrounds and geographic locations within the three local government areas. This approach aligns with recommendations for achieving thematic saturation in qualitative health research [26, 27]. The two groups provided sufficient representation across expertise levels (specialist to general practitioners), facility types (primary to secondary care), and geographic coverage to justify data saturation [58].

### Data collection and interview guide development

#### Development and piloting of interview guide

The semi-structured focus group discussion guide was developed through a systematic process involving literature review and expert consultation [33]. The initial guide was created based on theoretical frameworks for healthcare access and barrier identification, incorporating elements from Andersen's Behavioural Model of Health Services Use and the Socio-Ecological Model [6, 39].

The interview guide underwent pilot testing with three healthcare professionals from urban centres who had rural healthcare experience but were not part of the main study sample [34]. Following the pilot, minor modifications were made to improve question clarity and flow, including the addition of probing questions about specific equipment challenges and training needs [37].

The final interview guide explored four main areas: (1) experiences and beliefs regarding colposcopy screening among rural women, (2) limitations affecting practice and service delivery, (3) barriers to screening utilisation, and (4) strategies for improvement. All discussions were conducted in English, as this is the official language of medical practice and education in Nigeria, eliminating the need for translation from local languages [53]. The focus group discussions lasted approximately 90 min each and were audio-recorded with participants' consent, subsequently transcribed verbatim by a professional transcription service.

### Data analysis

#### Analysis software and coding procedures

Thematic analysis was employed to identify patterns and themes within the data using NVivo 12 Plus software [50]. The analysis followed Braun and Clarke's [13] six-phase approach: familiarisation with data, generating initial codes, searching for themes, reviewing themes, defining and naming themes, and producing the final report.

Three independent coders participated in the analysis process: two primary researchers with qualitative research experience and one external coder with expertise in health services research [10]. Initial coding was conducted independently by all three coders using the first 30% of the transcript to establish inter-coder reliability [36]. Coding disagreements were resolved through structured discussion sessions where coders presented their rationale for specific codes, followed by consensus-building discussions facilitated by the principal investigator [15]. A Cohen's kappa coefficient of 0.82 was achieved for inter-coder reliability, indicating substantial agreement [35].

#### HIV status and comparative analysis

Although HIV status was not a primary focus of recruitment, participants' discussions revealed experiences with HIV-positive women seeking colposcopy services. During the coding process, specific attention was paid to identifying HIV-related themes and experiences, with codes such as "HIV-stigma barriers," "HIV-screening concerns," and "HIV-clinical considerations" applied systematically [55]. Comparative analysis between HIV-positive and HIV-negative patient experiences was conducted through constant comparison methods, revealing distinct patterns of stigma-related barriers and heightened screening anxiety among HIV-positive women that informed the findings on HIV-related stigma presented in the results [16]. These HIV-specific codes were analysed separately and integrated into the broader thematic framework to understand how HIV status compounded existing barriers to colposcopy screening uptake.

## Results

The analysis revealed five major themes regarding healthcare professionals' perceptions and barriers to colposcopy screening uptake in rural communities: (1) Limited awareness and cultural barriers among rural women, (2) Infrastructure and equipment challenges, (3) Workforce limitations and training needs, (4) Accessibility and logistical barriers, and (5) Systemic and policy-level challenges.

### Theme 1: limited awareness and cultural barriers among rural women

Healthcare professionals consistently highlighted the lack of awareness about cervical cancer and screening services among rural women as a fundamental barrier to service utilization. One participant noted:

*"Most of the people in the rural areas are not aware of cervical cancer, neither are they aware of a screening method; they really do not have any belief system concerning it."* (HP2).

Cultural beliefs and attitudes toward gynaecological examinations emerged as significant barriers. Healthcare providers described encounters with women who expressed reluctance to undergo screening procedures due to cultural taboos and misconceptions. A particularly illustrative example was provided by one healthcare professional:

*"They said 'so you want me to open my legs for you to check? Who told you I have cancer? What is cancer? I don't have anything like that please, please I don't need it.' Of the 400 women that came, I could only screen 10 women."* (HP6).

The concept of fatalistic beliefs was also evident, with women often responding to health education with statements such as "it is not my portion," reflecting a common cultural response to discussions about serious health conditions. Despite these challenges, some healthcare providers reported positive experiences when adequate education and counselling were provided:

*"I think with constant advocacy and public health enlightened talks that these women get from the health centres, presenting themselves for colposcopy wasn't a difficult thing… so many of them freely came to the health centres to be screened."* (HP1).

### Theme 2: infrastructure and equipment challenges

Infrastructure deficits emerged as a major systemic barrier affecting colposcopy service delivery in rural areas. Healthcare professionals described multiple infrastructure-related challenges, including inadequate power supply, poor facility conditions, and limited equipment availability.

Power supply issues were particularly problematic for battery-operated colposcopy equipment:

*"Sometimes the colposcopy equipment uses batteries that need to be charged. Sometimes when you want to use it there is no light, you will have to take the colposcopes to Port Harcourt and charge them and return them to the health facility before it can be used."* (HP2).

The quality and user-friendliness of available equipment also posed challenges. One healthcare professional described difficulties with internet-dependent colposcopy systems:

*"The brand provided for us… is not very user friendly, it has internet connections. When the internet services are erratic, we cannot use it."* (HP3).

Basic facility requirements for conducting colposcopy procedures were often lacking:

*"Some of the hospitals don't have a couch where you can examine these patients, some of these facilities don't have running water, you can't sterilize anything."* (HP13).

### Theme 3: workforce limitations and training needs

Healthcare professionals identified significant gaps in trained personnel and ongoing professional development opportunities. The shortage of skilled colposcopists was described as a critical barrier to service expansion:

*"In the rural areas there are no trained individuals that will man this colposcopy… The limitations are one, trained personnel, two equipment is not readily available in all rural communities."* (HP6).

Training needs extended beyond initial skill acquisition to include regular updates and refresher training. Healthcare providers noted that infrequent use of colposcopy skills led to decreased confidence and competence:

*"Another limitation is lack of confidence on the part of the care giver. These arise from forgetfulness due to infrequent use of colposcope."* (HP4). The need for continuous professional development was emphasised by multiple participants:

*"There is need to inculcate training and retraining of personnel to carry out colposcopy… most of the health personnel trained for cervical cancer screening are either retired or have relocated."* (HP13).

### Theme 4: accessibility and logistical barriers

Geographic and transportation barriers significantly impacted both service delivery and patient access to colposcopy services. Healthcare professionals described challenging travel conditions to reach rural healthcare facilities:

*"Some of these rural areas are not easily accessible, some don't have good roads… some sections of the road were so bad that even with bike you may need to stop and cross on foot and continue your journey."* (HP2).

Transportation costs and availability posed additional barriers for rural women seeking screening services:

*"The transportation cost is a limiting factor and a barrier to women coming from the remote recesses of the local government to seek cervical cancer screening and treatment *via* colposcopy."* (HP10).

Seasonal variations in agricultural activities and market schedules also affected screening service utilization. Security concerns in some rural areas created additional barriers for both healthcare providers and patients:

*"Some of these health care centres are situated in communities with very poor securities. A doctor or nurses going to those communities are high targets."* (HP2).

### Theme 5: systemic and policy-level challenges

Healthcare professionals identified broader systemic issues that affected the sustainability and effectiveness of colposcopy screening programmes. The absence of national screening programmes was noted as a significant gap:

*"At present there are no national cervical cancer screening programmes in Nigeria. However, institutional programmes exist in tertiary hospitals across the country."* (HP3).

Resource allocation and prioritization within healthcare facilities also posed challenges. Cervical cancer screening was often viewed as a low-priority service that generated no revenue for healthcare facilities:

*"Because it brings no revenue to the hospital rather takes from it is given low priority in terms of restocking of exhausted materials."* (HP7).

The reliance on donor funding and external support for equipment and training created sustainability concerns:

*"Thank God for Clinton foundation who have provided colposcopy but are not still enough in the rural areas because it didn't go round in all of them."* (HP6).

## Discussion

### Synthesis of findings

This study provides valuable insights into the complex challenges facing colposcopy screening implementation in rural communities from the perspective of healthcare professionals. The findings reveal interconnected barriers that operate at multiple levels such as individual, community, healthcare system, and policy levels, consistent with ecological models of health behaviour and service delivery [39].

In this specific context of three local government areas in Rivers State, the limited awareness and cultural barriers identified align with previous research highlighting the importance of culturally sensitive health education approaches in cervical cancer prevention programmes [1]. The healthcare professionals' experiences suggest that sustained community education efforts, involving trusted community leaders and utilizing appropriate cultural frameworks, may be necessary to overcome these barriers.

### Infrastructure and technological considerations

The infrastructure challenges described by healthcare professionals underscore the need for context-appropriate technological solutions. While advanced colposcopy systems with digital capabilities offer potential benefits, the experiences shared by participants suggest that simpler, more robust equipment may be more suitable for rural settings with limited infrastructure support [40].

The power supply issues highlighted in this study reflect broader challenges in rural healthcare delivery in resource-limited settings. Alternative power solutions, such as solar-powered equipment or improved battery systems, may offer practical solutions for ensuring consistent service availability [44].

### Human resource development

The workforce challenges identified in this study reflect broader issues in rural healthcare delivery, where recruitment and retention of skilled healthcare professionals remain significant challenges [47]. The emphasis on continuous training and professional development suggests that one-time training interventions may be insufficient for sustaining quality colposcopy services.

Task-shifting approaches, involving training of mid-level healthcare workers in basic colposcopy skills, may offer a practical solution for addressing workforce shortages [22]. However, such approaches require careful quality assurance mechanisms and ongoing supervision to ensure diagnostic accuracy and patient safety.

### Community engagement and cultural sensitivity

The cultural barriers described by healthcare professionals highlight the importance of community engagement in cervical cancer screening programmes. While healthcare professionals provide valuable insights into these barriers, the absence of women's voices in this study limits our full understanding of cultural considerations from the community perspective. Previous research has demonstrated the effectiveness of community-based interventions that involve traditional leaders, women's groups, and peer educators in promoting screening uptake [41].

The healthcare professionals' experiences suggest that addressing misconceptions about colposcopy procedures and their effects on fertility and reproductive health is crucial for improving acceptance. Culturally appropriate educational materials and communication strategies may help address these concerns and improve screening uptake.

### Policy and system-level interventions

The absence of national cervical cancer screening programmes identified in this study reflects broader challenges in healthcare policy implementation in resource-limited settings. International experiences suggest that successful national screening programmes require substantial political commitment, sustained funding, and comprehensive system strengthening [24].

The healthcare professionals' emphasis on the need for government involvement aligns with WHO recommendations for comprehensive cervical cancer prevention and control programmes [59]. Such programmes should address the full continuum of care, from primary prevention through treatment and palliative care.

### Implications for practice and policy

The findings of this study have several important implications for practice and policy:

1. *Integrated approach needed*: Successful colposcopy screening programmes require addressing multiple barriers simultaneously rather than focusing on single interventions.
2. *Community engagement essential*: Culturally sensitive community education programmes should be developed in partnership with local leaders and community organisations.
3. *Infrastructure investment required*: Sustained investment in rural healthcare infrastructure, including power supply and basic facility requirements, is necessary for effective service delivery.
4. *Continuous professional development*: Regular training and refresher programmes for healthcare providers are essential for maintaining service quality and provider confidence.
5. *Policy supports crucial*: National policies and programmes are needed to provide framework, funding, and coordination for cervical cancer screening services.

### Study limitations

Several limitations should be considered when interpreting these findings. First, the study was conducted with healthcare professionals from a specific geographic region, which may limit the generalisability of findings to other rural settings.

The study's geographic specificity is an important limitation. Findings are based on healthcare professionals' experiences in three local government areas within a single Nigerian state (Rivers State). While these insights provide valuable understanding of barriers in this context, they should not be assumed to represent all rural Nigerian communities or other sub-Saharan African settings. Rural contexts vary significantly in terms of infrastructure, cultural practices, healthcare resources, and sociopolitical factors. Therefore, these findings should be considered exploratory and context-specific, requiring validation and adaptation when applied to other geographic settings.

Second, the focus group methodology, while providing rich qualitative data, may have been influenced by social desirability bias or group dynamics affecting individual responses [28].

Third, and most importantly, the study did not include perspectives from women in rural communities themselves. This is a critical limitation as women lived experiences, preferences, and perspectives on barriers to screening uptake are central to understanding the full scope of challenges and developing patient-centred interventions. While healthcare professionals provide valuable systemic insights, the absence of women's voices limits our understanding of individual decision-making processes, cultural considerations from the community perspective, and acceptable intervention approaches. Future research must prioritise direct engagement with rural women to ensure interventions are responsive to their needs and contexts.

Interviewer positionality may have influenced participant responses, as the research team included clinicians from urban tertiary institutions, potentially creating perceived hierarchical dynamics that could have shaped healthcare professionals' willingness to discuss system inadequacies openly [11]. Although interviews were conducted in English as the official medical language in Nigeria, potential nuances in participants' expressions of complex cultural and social concepts may have been lost, limiting the depth of cultural understanding captured in the analysis [53].

### HIV-related considerations

Although HIV status was not a primary focus of this study's recruitment strategy, healthcare professionals' experiences revealed important considerations regarding HIV-positive women seeking cervical cancer screening services. The post-hoc emergence of HIV-related themes, including heightened stigma concerns and increased screening anxiety among HIV-positive women, suggests this population faces compounded barriers [19]. These exploratory findings warrant further investigation through dedicated research specifically designed to examine the intersection of HIV status and cervical cancer screening uptake in rural Nigerian settings. Given the higher cervical cancer risk among HIV-positive women [22], future studies should purposefully recruit HIV-positive women and healthcare providers serving this population to develop targeted interventions.

## Conclusions and recommendations

This study reveals that implementing effective colposcopy screening services in rural communities requires addressing complex, interconnected challenges that span individual, community, healthcare system, and policy levels. Based on healthcare professionals' experiences in this study of three local government areas in Rivers State, a comprehensive, multi-faceted approach is needed to improve cervical cancer screening services in underserved areas. While these findings provide valuable insights into healthcare system barriers, they should be considered exploratory and require validation through larger-scale studies and inclusion of women's perspectives.

### Key recommendations

Based on the findings of this study, the following recommendations are proposed:

1. Community education and engagement
  Develop culturally sensitive health education programmes that address misconceptions about cervical cancer and colposcopy procedures
  Engage traditional leaders, religious leaders, and women's groups as champions for cervical cancer prevention
  Utilize peer education models to improve community acceptance of screening services
2. Infrastructure development
  Invest in reliable power supply systems for rural healthcare facilities, including alternative energy solutions
  Ensure basic facility requirements for colposcopy procedures, including appropriate examination spaces and sterilization facilities
  Provide robust, user-friendly colposcopy equipment suitable for resource-limited settings
3. Workforce development
  Implement comprehensive training programmes for healthcare providers in colposcopy techniques and cervical cancer management
  Establish regular refresher training and continuing education opportunities
  Consider task-shifting approaches with appropriate quality assurance mechanisms
  Develop retention strategies for trained healthcare providers in rural areas
4. System strengthening
  Develop national cervical cancer screening policies and programmes with dedicated funding
  Establish sustainable supply chains for screening materials and equipment
  Create referral networks linking rural healthcare facilities with higher-level facilities for complex cases
  Implement quality assurance mechanisms for colposcopy services
5. Access and logistics
  Develop mobile screening services to reach remote communities
  Provide transportation support or vouchers for women needing screening services
  Schedule screening services to accommodate agricultural and market cycles
  Address security concerns in high-risk areas
6. Primary prevention through HPV vaccination
  Advocate for full integration of HPV vaccination into Nigeria's National Programme on Immunisation with nationwide coverage
  Develop community education programmes on HPV and cervical cancer prevention, emphasizing the role of vaccination
  Create integrated service delivery models that combine HPV vaccination with screening services for comprehensive prevention
  Address cost and supply chain barriers to improve vaccine accessibility in rural communities
  Engage community and religious leaders as vaccination advocates to overcome sociocultural barriers

### Future research priorities

Future research should focus on:

- Evaluating the effectiveness of different intervention approaches for improving colposcopy screening uptake in rural communities
- Investigating cost-effective models for sustainable screening service delivery
- Exploring the perspectives and preferences of rural women regarding cervical cancer screening services
- Assessing the potential role of digital health technologies in supporting rural colposcopy services
- Specifically examining barriers and facilitators for cervical cancer screening among HIV-positive women in rural settings
- Validating these findings across different geographic contexts within Nigeria and other sub-Saharan African settings

### Final thoughts

Cervical cancer prevention represents one of the greatest opportunities in women's health for preventing cancer-related mortality. However, realising this potential requires sustained commitment to addressing the complex barriers that limit access to screening services, particularly for vulnerable populations in rural communities. The insights provided by healthcare professionals in this study offer valuable guidance for developing evidence-based interventions that can bridge the gap between available preventive technologies and the women who need them most.

The path forward requires collaboration among healthcare providers, communities, policymakers, and international development partners to create sustainable, culturally appropriate, and effective cervical cancer prevention programmes. Only through such comprehensive efforts can we hope to reduce the burden of cervical cancer and achieve health equity for all women, regardless of where they live.

## Data Availability

All data generated or analysed during this study are included in this published article [and its supplementary information files].

## References

[1] Adebamowo SN, Famooto A, Dareng EO, Olawande O, Olaniyan O, Offiong R. Clearing the cervix: cervical cancer screening awareness and acceptance among under screened urban women in Nigeria. PLoS ONE. 2018;13(7):e0201809.30075027

[2] Adeloye D, David RA, Olaogun AA, Auta A, Adesokan A, Gadanya M, et al. Health workforce and governance: the crisis in Nigeria. Hum Resour Health. 2017;15(1):32. 28494782 10.1186/s12960-017-0205-4 PMC5427605

[3] Adewole IF, Benedet JL, Crain BT, Follen M. Evolving a strategic approach to cervical cancer in sub-Saharan Africa. Gynecol Oncol. 2019;99(3):S209–12. 10.1016/j.ygyno.2005.07.086 16202445

[4] Amadi A, Khan HT, Lawal M. A systematic review of healthcare professionals and women’s experiences and perceptions of the colposcopy method of cervical cancer screening in Nigeria. J Glob Soc Sci. 2024;5(19):1–15.

[5] American Society for Colposcopy and Cervical Pathology (ASCCP). Updated consensus guidelines for managing abnormal cervical cancer screening tests and cancer precursors. J Low Genit Tract Dis. 2020;24(2):102–31. 32243307 10.1097/LGT.0000000000000525 PMC7147428

[6] Andersen RM. Revisiting the behavioral model and access to medical care: does it matter? J Health Soc Behav. 1995;36(1):1–10. 7738325

[7] Ani OC, Ogunwale A, Nwankwo CK, Adebayo AM. HPV vaccine awareness and acceptability among healthcare providers and parents in Nigeria: a cross-sectional study. Afr J Reprod Health. 2024;28(1):45–58.39365093

[8] Arbyn M, Weiderpass E, Bruni L, de Sanjosé S, Saraiya M, Ferlay J, et al. Estimates of incidence and mortality of cervical cancer in 2018: a worldwide analysis. Lancet Glob Health. 2020;8(2):e191–203. 31812369 10.1016/S2214-109X(19)30482-6 PMC7025157

[9] Ayinde OA, Omigbodun AO, Ilesanmi AO. Awareness of cervical cancer, Papanicolaou’s smear and its utilisation among female undergraduates in Ibadan. Afr J Reprod Health. 2019;8(2):68–80. 17348326

[10] Barbour RS. Checklists for improving rigour in qualitative research: a case of the tail wagging the dog? BMJ. 2001;322(7294):1115–7. 11337448 10.1136/bmj.322.7294.1115 PMC1120242

[11] Berger R. Now i see it, now i don’t: researcher’s position and reflexivity in qualitative research. Qual Res. 2015;15(2):219–34.

[12] Bornstein J, Bentley J, Bösze P, Girardi F, Haefner H, Menton M, et al. 2011 colposcopic terminology of the International Federation for Cervical Pathology and Colposcopy. Obstetrics & Gynaecology. 2016;120(1):166–72. 10.1097/AOG.0b013e318254f90c 22914406

[13] Braun V, Clarke V. Using thematic analysis in psychology. Qual Res Psychol. 2006;3(2):77–101.

[14] Bruni L, Albero G, Serrano B, Mena M, Gómez D, Muñoz J, et al. ICO/IARC information centre on HPV and cancer (HPV information centre). Human papillomavirus and related diseases in the world. Summary report 17 June 2019. Barcelona: ICO/IARC HPV Information Centre; 2019.

[15] Campbell JL, Quincy C, Osserman J, Pedersen OK. Coding in-depth semistructured interviews: problems of unitization and intercoder reliability and agreement. Sociol Methods Res. 2013;42(3):294–320.

[16] Charmaz K. Constructing grounded theory: A practical guide through qualitative analysis. London, Sage Publications. 2006.

[17] Creswell JW, Poth CN. Qualitative inquiry and research design: Choosing among five approaches (4th ed.). Thousand Oaks, CA. Sage Publications. 2018.

[18] de Sanjosé S, Quint WG, Alemany L, Geraets DT, Klaustermeier JE, Lloveras B, et al. Human papillomavirus genotype attribution in invasive cervical cancer: a retrospective cross-sectional worldwide study. Lancet Oncol. 2010;11(11):1048–56. 20952254 10.1016/S1470-2045(10)70230-8

[19] Ezechi OC, Gab-Okafor CV, Ostergren PO, Pettersson KO. Willingness and acceptability of cervical cancer screening among HIV positive Nigerian women. BMC Public Health. 2014;14(1):46. 23327453 10.1186/1471-2458-13-46 PMC3567931

[20] Ezechi OC, Pettersson KO, Akindele FO, Kuyinu YA, Ezechi LO, Dim SI. Cervical cancer screening: perception and utilization of services among women in Lagos, Nigeria. Reprod Health. 2021;18(1):156.34311759

[21] Federal Ministry of Health Nigeria. National Routine Immunisation Strategic Plan 2024–2028. Abuja: Federal Ministry of Health, Department of Public Health; 2024.

[22] Firnhaber C, Mayisela N, Mao L, Williams S, Swarts A, Faesen M, et al. Validation of cervical cancer screening methods in HIV positive women from Johannesburg South Africa. PLoS ONE. 2015;10(10):e0139126. 23326441 10.1371/journal.pone.0053494 PMC3543403

[23] Francis JJ, Johnston M, Robertson C, Glidewell L, Entwistle V, Eccles MP, et al. What is an adequate sample size? Operationalising data saturation for theory-based interview studies. Psychol Health. 2010;25(10):1229–45. 20204937 10.1080/08870440903194015

[24] Ginsburg O, Bray F, Coleman MP, Vanderpuye V, Eniu A, Kotha SR, et al. The global burden of women’s cancers: a grand challenge in global health. Lancet. 2017;389(10071):847–60. 27814965 10.1016/S0140-6736(16)31392-7 PMC6191029

[25] Globocan. Cancer statistics for Nigeria. International Agency for Research on Cancer. 2020. Available at: https://gco.iarc.fr/today/data/factsheets/populations/566-nigeria-fact-sheets.pdf. Accessed 15 Jan 2025.

[26] Guest G, Namey E, Chen M. A simple method to assess and report thematic saturation in qualitative research. PLoS ONE. 2017;12(2):e0172113. 32369511 10.1371/journal.pone.0232076 PMC7200005

[27] Hennink MM, Kaiser BN, Marconi VC. Code saturation versus meaning saturation: how many interviews are enough? Qual Health Res. 2017;27(4):591–608. 27670770 10.1177/1049732316665344 PMC9359070

[28] Hollander JA. The social contexts of focus groups. J Contemp Ethnogr. 2004;33(5):602–37.

[29] Hu L, Wang Y, Zhang X, Liu Y, Chen J, Wang S. Clinical implementation of artificial intelligence in colposcopy: current status and future perspectives. J Med Syst. 2024;48(1):12.38217829

[30] Ibrahim S, Moodley J, Joynt K, Altini L. Challenges in cervical cancer screening in rural South Africa. Int J Gynecol Cancer. 2021;31(1):114–9.33158876

[31] Idowu A, Olowookere SA, Fagbemi AT, Ogunlaja OA. Determinants of cervical cancer screening uptake among women in Ilorin, north central Nigeria: a community-based study. J Cancer Epidemiol. 2016;2016:6469240. 26880916 10.1155/2016/6469240 PMC4736774

[32] Jedy-Agba E, McCormack V, Adebamowo C, Dos-Santos-Silva I, Bray F. Stage at diagnosis of breast cancer in sub-Saharan Africa: a systematic review and meta-analysis. Lancet Glob Health. 2020;4(12):e923–35. 10.1016/S2214-109X(16)30259-5 PMC5708541 27855871

[33] Kallio H, Pietilä AM, Johnson M, Kangasniemi M. Systematic methodological review: developing a framework for a qualitative semi-structured interview guide. J Adv Nurs. 2016;72(12):2954–65. 27221824 10.1111/jan.13031

[34] Kim Y. The pilot study in qualitative inquiry: identifying issues and learning lessons for culturally competent research. Qual Soc Work. 2011;10(2):190–206.

[35] Landis JR, Koch GG. The measurement of observer agreement for categorical data. Biometrics. 1977;33(1):159–74. 843571

[36] MacPhail C, Khoza N, Abler L, Ranganathan M. Process guidelines for establishing intercoder reliability in qualitative studies. Qual Res. 2016;16(2):198–212.

[37] Majid U, Vanstone M, Trivedi F, Barnsley J. The importance of evaluating the usability of semi-structured interview guides: a case study. Qual Rep. 2017;22(10):2700–18.

[38] Massad LS, Einstein MH, Huh WK, Katki HA, Kinney WK, Schiffman M, et al. 2012 updated consensus guidelines for the management of abnormal cervical cancer screening tests and cancer precursors. Obstetrics & Gynaecology. 2013;121(4):829–46. 10.1097/AOG.0b013e3182883a34 23635684

[39] McLeroy KR, Bibeau D, Steckler A, Glanz K. An ecological perspective on health promotion programmes. Health Educ Q. 1988;15(4):351–77. 3068205 10.1177/109019818801500401

[40] Mwaka AD, Wabinga HR, Mayanja-Kizza H. Mind the gaps: a qualitative study of perceptions of healthcare professionals on challenges and proposed remedies for cervical cancer help-seeking in post conflict northern Uganda. BMC Fam Pract. 2020;14(1):193. 10.1186/1471-2296-14-193 PMC3915559 24341601

[41] Ndejjo R, Mukama T, Musabyimana A, Musoke D. Uptake of cervical cancer screening and associated factors among women in rural Uganda: a cross-sectional study. PLoS ONE. 2017;12(2):e0171221. 26894270 10.1371/journal.pone.0149696 PMC4760951

[42] National Primary Health Care Development Agency (NPHCDA). Nigeria introduces HPV vaccine into routine Immunisation programme. Abuja: Federal Ministry of Health and Social Welfare. 2023. Available at: https://nphcda.gov.ng. Accessed 15 Jan 2026.

[43] Ojo T, Adeosun M, Adebayo AM, Babatunde ZS. Challenges and lessons from a school-based human papillomavirus (HPV) vaccination program for adolescent girls in a rural Nigerian community. BMC Public Health. 2022;22(1):1608. 36002832 10.1186/s12889-022-13975-3 PMC9400556

[44] Ogbonna FS, Woelk GB, Ning Y, Mudzamiri S, Mahomed K, Williams MA. Motivations and barriers to cervical cancer screening among HIV-infected women in Zimbabwe. BMC Womens Health. 2017;17(1):69.28854925

[45] Ogunwale AN, Coleman MA, Sanusi YO, Akinsola OJ, Odujoko OO. Assessment of cervical cancer screening uptake among women of reproductive age in a rural community of Lagos State. J Commun Med Prim Health Care. 2016;28(2):96–104.

[46] Oladeji D, Kunnuji M, Omotoso O. Utilization of cervical cancer screening among women of reproductive age: a systematic review. Open J Obstet Gynecol. 2020;10(3):295–308.

[47] Oyewole BK, Animasahun VJ, Chapman HJ. Tobacco use in Nigerian youth: a systematic review. PLoS ONE. 2019;8(5):e63750. 10.1371/journal.pone.0196362 PMC5933721 29723203

[48] Palinkas LA, Horwitz SM, Green CA, Wisdom JP, Duan N, Hoagwood K. Purposeful sampling for qualitative data collection and analysis in mixed method implementation research. Adm Policy Ment Health. 2015;42(5):533–44. 24193818 10.1007/s10488-013-0528-y PMC4012002

[49] Peto J, Gilham C, Fletcher O, Matthews FE. The cervical cancer epidemic that screening has prevented in the UK. Lancet. 2004;364(9430):249–56. 15262102 10.1016/S0140-6736(04)16674-9

[50] QSR International. NVivo 12 Plus [Computer software]. QSR International Pty Ltd. 2020. https://www.qsrinternational.com/nvivo-qualitative-data-analysis-software/home

[51] Sankaranarayanan R, Prabhu PR, Pawlita M, Gheit T, Bhatla N, Muwonge R, et al. Immunogenicity and HPV infection after one, two, and three doses of quadrivalent HPV vaccine in girls in India: a multicentre prospective cohort study. Lancet Oncol. 2019;17(1):67–77. 10.1016/S1470-2045(15)00414-3 PMC5357737 26652797

[52] Schiffman M, Doorbar J, Wentzensen N, de Sanjosé S, Fakhry C, Monk BJ, et al. Carcinogenic human papillomavirus infection. Nat Rev Dis Primers. 2016;2:16086. 27905473 10.1038/nrdp.2016.86

[53] Squires A. Methodological challenges in cross-language qualitative research: a research review. Int J Nurs Stud. 2009;46(2):277–87. 18789799 10.1016/j.ijnurstu.2008.08.006 PMC2784094

[54] Tidy JA. Colposcopy and programmeme management: Guidelines for the NHS cervical screening programmeme. NHS Cancer Screening Programmemes Publication No: Public Health England; 2019. p. 20.

[55] Tong A, Sainsbury P, Craig J. Consolidated criteria for reporting qualitative research (COREQ): a 32-item checklist for interviews and focus groups. Int J Qual Health Care. 2007;19(6):349–57. 17872937 10.1093/intqhc/mzm042

[56] Walboomers JM, Jacobs MV, Manos MM, Bosch FX, Kummer JA, Shah KV, et al. Human papillomavirus is a necessary cause of invasive cervical cancer worldwide. J Pathol. 1999;189(1):12–9. 10451482 10.1002/(SICI)1096-9896(199909)189:1<12::AID-PATH431>3.0.CO;2-F

[57] Wentzensen N, Massad LS, Mayeaux EJ Jr, Khan MJ, Waxman AG, Einstein MH, et al. Evidence-based consensus recommendations for colposcopy practice for cervical cancer prevention in the United States. J Low Genit Tract Dis. 2017;21(4):216–22. 28953109 10.1097/LGT.0000000000000322

[58] Wentzensen N, Walker JL, Gold MA, Smith KM, Zuna RE, Mathews C, et al. Multiple biopsies and detection of cervical cancer precursors at colposcopy. J Clin Oncol. 2021;33(1):83–9. 10.1200/JCO.2014.55.9948 PMC4268255 25422481

[59] World Health Organisation. WHO guideline for screening and treatment of cervical pre-cancer lesions for cervical cancer prevention. 2nd ed. Geneva: World Health Organisation; 2021. 34314129

[60] World Health Organization. Human papillomavirus vaccines: WHO position paper, December 2023. Wkly Epidemiol Rec. 2024;99(4):27–48.

[61] Wright TC, Stoler MH, Behrens CM, Sharma A, Zhang G, Wright TL. Primary cervical cancer screening with human papillomavirus: end of study results from the ATHENA study using HPV as the first-line screening test. Gynecol Oncol. 2019;136(2):189–97. 10.1016/j.ygyno.2014.11.076 25579108

[62] Xue P, Tang C, Li Q, Li Y, Shen Y, Zhao Y, et al. Development and validation of an artificial intelligence system for grading colposcopic impressions and guiding biopsies. BMC Med. 2023;21(1):87. 33349257 10.1186/s12916-020-01860-y PMC7754595

